# Lactate Sensors on Flexible Substrates

**DOI:** 10.3390/bios6030048

**Published:** 2016-09-21

**Authors:** Xuesong Yang, Timothy Fu, Pavan Kumar Kota, Maggie Tjia, Cuong Manh Nguyen, Jung-Chih Chiao

**Affiliations:** 1 Electrical Engineering, University of Texas‒Arlington, Arlington, TX 76019, USA; pavankumar.kota@mavs.uta.edu (P.K.K.); maggie.tjia@mavs.uta.edu (M.T.); nmanhcuong@gmail.com (C.M.N.); jcchiao@uta.edu (J.-C.C.); 2 Texas A&M Health Science Center, Bryan, TX 77807, USA; timwfu@gmail.com

**Keywords:** flexible substrate, lactate, IrO_x_, biosensor

## Abstract

Lactate detection by an in situ sensor is of great need in clinical medicine, food processing, and athletic performance monitoring. In this paper, a flexible, easy to fabricate, and low-cost biosensor base on lactate oxidase is presented. The fabrication processes, including metal deposition, sol-gel IrO_x_ deposition, and drop-dry enzyme loading method, are described in detail. The loaded enzyme was examined by scanning electron microscopy. Cyclic voltammetry was used to characterize the sensors. Durability, sensibility, and selectivity of the biosensors were examined. The comparison for different electrode sizes and different sensing film materials was conducted. The sensor could last for four weeks with an average surface area normalized sensitivity of 950 nA/(cm^2^ mM) and 9250 nA/(cm^2^ mM) for Au-based electrodes, and IrO_x_-modified electrodes respectively, both with an electrode size of 100 × 50 μm. The self-referencing method to record noises simultaneously with the working electrode greatly improved sensor sensitivity and selectivity. The sensor showed little response to interference chemicals, such as glutamate and dopamine.

## 1. Introduction

Lactate is a common analyte due to its wide variety of applications. In the field of food processing, L-lactate is present in the fermentation of cheese, yoghurt, butter, pickles, sauerkraut, and other food products [1,2]. Monitoring lactate concentrations can be used to assess the condition of freshness in dairy products [1]. In fish farming, health and stress levels of fish can be monitored by testing the lactic acid concentrations, thereby limiting the mass use of antibiotics and the inadvertent consequences by human consumption of the residual antibiotics [3,4].

In human bodies, lactate is metabolized predominantly in the kidney and liver [5]. The normal lactate level range in the human body is 0.5‒2.0 mM/L [6,7]. Hyperlactatemia is defined when lactate levels are between 2 mM/L (millimol/liter) to 5 mM/L [8,9]. When lactate levels exceed 5 mM/L, the conditions indicate severe lactic acidosis [10]. In the fields of human performance monitoring, lactate levels play a key role to brain blood flow and have an impact on brain activation during exercise-induced fatigue [5]. Transient lactic acidosis can occur due to excessive lactate production from tissue hypoxia or increased cellular metabolism caused by strenuous exercise [10,11,12]. By monitoring the lactate thresholds in endurance athletes, a lactate sensor can inform users of their limits during exercise [5].

In clinical medicine, hyperlactataemia is an indicator of systemic tissue dysoxia or abnormal microcirculatory perfusion [13]. It may also indicate the severity liver injury and the accompanying multiple organ failure [11,14]. Lactic acidosis is indicative of tissue ischemia, liver disease, kidney disease, sepsis, and shock [12]. Persisting lactic acidosis may indicate an issue with hepatic metabolism in which the lactate production exceeds the rate the liver can metabolize [14]. Persisting high blood lactate concentration is associated with poor prognosis in patient mortality [6]. Measuring blood lactate accurately enables clinicians with an early-prediction prognosis [15]. High levels of local lactate within head, neck and uterine tumor cells may be associated with a greater risk of cancer metastasis [6]. Measuring lactate levels may lead to differentiating between metastatic and benign tumors in those regions [6,7].

In clinical practice, blood lactate levels can be measured using central laboratory test equipment, point-of-care blood gas analyzers, spectrophotometric analyzers, and emerging hand-held devices [16,17,18]. The conventional analyzers have certain disadvantages, such as the large size, heavy weight, lack of deformability, and complexity of operation. Techniques, such as indirect spectrophotometry, liquid chromatography, and magnetic spectroscopy [19,20,21], require the use of sophisticated equipment and the systems could be expansive, which are unsuitable for wearable, implantable, or disposable applications. With the increasing needs for portable devices, varieties of techniques have been presented. Baker and Gough have demonstrated a modified platinum and silver wire electrode with the pore-free silicon rubber insulation layer. The result showed a sensitivity of 2.5 nA/(cm^2^ mM) [22]. Schabmueller et al. demonstrated a micromachined lactate sensor using a titanium-latinum working electrode and titanium-platinum-iridium oxide reference electrode on a double-sided silicon-on-insulator wafer [23]. Burmeister et al. demonstrated a three-layered microelectrode array using Nafion and polyurethane on ceramic. The result showed a detection limit of 0.078 ± 0.013 mM [24]. Multi-site sensors have been investigated to monitor lactate and glucose, among other parameters. Perdomo et al. have demonstrated a multi-enzyme sensor with flow channels on a microfabricated silicon chip for real-time monitoring [25]. Elie et al. demonstrated an amperometric sensor with arrayed platinum electrodes on a glass substrate, with linearity up to 90 mM lactate [26]. Kurita et al. introduced a microfluidic device with carbon film electrodes on glass plates [27]. The result showed a lactate detection limit of 2.3 μM.

Deformability, wireless data transduction, and low power consumption are essential for biomedical in vivo applications. Sensors with rigid substrates, such as silicon, ceramic, and glass, have the disadvantages of being brittle and creating tissue damage when inserted into tissues. To address the needs, polymer-based flexible sensors have been proposed. Revzin et al. have demonstrated a lactate/glucose/pyruvate sensor array using deposited gold electrodes on a Mylar substrate. The result showed a lactate sensitivity of 240 nA/(cm^2^ mM) [28]. Weltin et al. have demonstrated a lactate and glutamate sensor using a deposited platinum/titanium electrode on polyimide foil [29]. In this work different membranes were studied to overcome the weak bounding problem between the enzyme and the metal electrode, which commonly exists for surface-type sensors [29]. Labroo and Cui have demonstrated a graphene-based lactate nanosensor on a flexible polyester film. The result showed a detection range of 0.08‒20 μM [30]. Jia et al. have demonstrated a skin-worn lactate sensor utilizing screen-printed carbon electrodes on temporary transfer “tattoo-base” paper. The result showed lactate sensing with linearity up to 20 mM [31]. Khodagholy et al. have demonstrated an organic electrochemical transistor for lactate detection with an ionogel solid-state electrolyte [32].

In this work, we propose a flexible, lightweight, micro-sized lactate sensor which can be integrated to our previously demonstrated wearable wireless module for data transduction [33]. The sensor configuration and the fabricated procedures have been simplified to allow low-power amplifier integration and reduce fabrication cost. A dual-electrode sensor based on lactate oxidase (LO_x_) has been fabricated and measured. The self-referencing method [34] was applied to eliminate the interference of similar chemicals, such as glutamate and dopamine, as well as noises from other conductive ions. To further improve the sensor performance, we modify the working electrode with the inert and bio-compatible material iridium oxide. Cox and Lewinski have demonstrated the use of IrOx in hydrogen peroxide detection [35]. Schabmueller et al. have used IrO_x_ as reference electrode [23]. In our previous work, we used IrO_x_ for pH detection [34]. However IrO_x_ has never been used as the base electrodes for lactate detection before. Based on the literature, the capability of delivering a higher charge density makes IrO_x_ an attractive electrode material [36,37]. A reachable charge capacity of 4 mC/cm^2^ is much higher than common metals, such as platinum and gold, which have the charge capacities of a few tens of µC/cm^2^ [37]. Additionally, during charge deliveries IrO_x_ can eliminate the side-effects, such as gas evolution and metal corrosion, which commonly exist in metals [37]. Furthermore, IrO_x_ film was expected to have a rougher surface profile than gold, which provides a larger expanded reactive area for enzyme loading. Hence, we proposed modifying the sensing surface with IrO_x_ to improve the sensor performance.

There are several methods to fabricate iridium oxide films. The sputtering deposition method requires a costly target [38]. The thermal oxidation method requires a high temperature range [38]. The electro-deposition method requires a stable power supply system to control the film thickness and quality [39]. Hence, we chose the sol-gel process to fabricate iridium oxide [34], which is easy to conduct without high thermal budgets. Our flexible lactate sensor performed with good sensitivity, selectivity, and flexibility, without having to use membrane or cross-link materials. The deformable capability enables the sensor to be used on the skin or in vivo. The simpler, low-cost, and time-efficient fabrication method makes it possible to apply to disposable devices. The sensor could be used for a variety of test-and-dispose applications, such as food processing tests, daily ex vivo tests for patients, infants, and seniors, and emergency response use, without causing secondary pollution to the subjects.

## 2. Materials and Methods

### 2.1. Materials

Lactate oxidase (Pediococcus species), L-glutamic salt, and 3-hydroxytyramine hydrochloride were used (Sigma-Aldrich, St. Louis, MO, USA). L-lactic acid (lithium salt) 99% was obtained from Fisher Scientific. 1.3 mg LO_x_ was dissolved in 500 µL 1× phosphate buffer (PBS) solution to form the lactate oxidase enzyme stock solution [40]. Slight agitation was needed to accelerate the dissolution. Then the stock solution was aliquoted to 20 µL and stored at −20 °C. 960 mg L-lactic acid (lithium salt) was dissolved in 10 ml 10× PBS to form 1 M L-lactate stock solution. The stock solution was stored at room temperature. Thirty-eight milligrams of 3-hydroxytyramine hydrochloride was dissolved in 200 mL deionized (DI) water to form the 1 mM dopamine (DA) stock solution. L-glutamic salt (37.428 mg) was dissolved in 200 mL DI water to form the 1-mM glutamate (Glu) stock solution.

### 2.2. Gold-Electrode Device Fabrication

**Figure 1:**
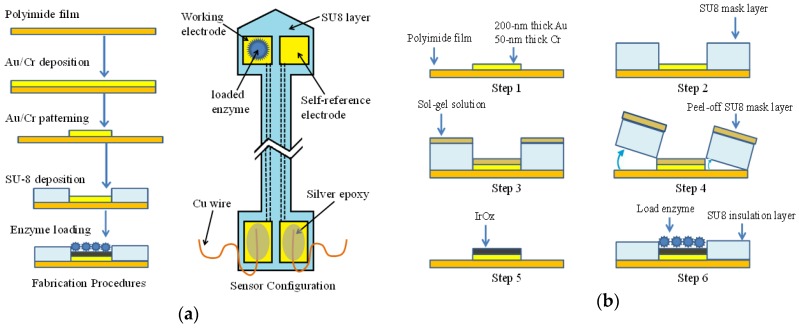
Fabrication process and sensor configuration: (**a**) gold electrode fabrication procedures and the sensor configuration; and (**b**) IrO_x_-modified electrode fabrication procedures.

The sensor probe was fabricated on a 125 µm thick flexible polyimide film. The substrate was cleaned by acetone and dried. An electron-beam deposition process was performed to pattern a layer of 200 nm thick gold on 50 nm thick chromium. Photolithography and wet-etching were carried out to pattern the probes with different electrode sizes of 1000 × 1000 µm, 500 × 500 µm, and 100 × 50 µm. A self-reference electrode (SE) was patterned next to the working electrode (WE) with the identical size and configuration. A second photolithography process was performed to form an insulation and protection layer. The samples were covered by SU8-25 (MicroChem) with open windows of sensing and contact pads for WE and SE. Each sensor contained one WE and one SE close to each other. The substrate with electrodes and connection lines was tailored by a sharp blade to a shaft shape that allowed the electrodes to be immersed in solution while the contact pads stayed above the liquid. Copper wires were attached to the contact pads by silver epoxy (Arctic Silver). The WE was loaded with LO_x_ enzymes afterwards. The step-by-step fabrication processes and the top view of the sensor are shown in Figure 1a. The entire sensor was covered by an SU-8 encapsulation layer while the two sensing pads and two connection pads were exposed. The WE was deposited with lactate oxidase, while the SE was not. The dashed lines indicate the metal lines underneath the insulation layer connecting the sensing electrodes and contact pads. Silver epoxy was used to fix copper wires which were connected to the measurement instruments.

### 2.3. I_r_O_x_-Electrode Device Fabrication

The IrO_x_ modified sensor fabrication process is shown in Figure 1b. After metal deposition, a thick layer of SU8-100 (MicroChem) was spin-coated and patterned to form a micro-channel structure on the top surface. A sol-gel process was conducted by dip-coating the sensor in the sol-gel solution (1 g iridium, 42 mL 95% ethanol, 10 ml 80% acetic acid). The amount of iridium accumulated on the surface was proportional to the depth of the SU8 well. However, the SU8-100 layer became brittle with the increase of thickness and the pattern was easily damaged during fabrication. Several different thicknesses had been tested and a thickness of 100 µm was preferred. After dip-coating, a layer of iridium mixture was formed on the patterned substrate, followed by a 20 min soft-bakeat 75 °C to remove the moisture. Then the flexible polyimide substrate was bent to peel off the SU8-100 layer. Afterwards, a thermal treatment was conducted to oxidize the iridium with a heating profile from 25 °C to 325 °C in a 3 h period. Then the temperature was maintained at 325 °C for 4 h, before cooling down in a 7 h period. An SU8-25 insulation layer was patterned over the metal patterns and cooper wires were connected to the contact pads. Detailed information for the sol-gel process can be found in our previous work [34]. Finally, lactate enzyme stock solution was loaded on the I_r_O_x_ sensing film.

### 2.4. LO_x_ Coating and Working Principle

To load the enzymes, the frozen LO_x_ stock solution was kept at room temperature for half an hour until completely thawed. The stock solution was gently agitated by a syringe tip to restore uniformity. The electrodes were cleaned by DI water and dried by air. Ten microliters of stock solution was transferred by a Hamilton syringe and deposited onto the electrode under a stereomicroscope. The same enzyme coating process was repeated four times. The sensors were sealed in a container and kept at the room temperature for two days before tests. During this period of time the protein was cured completely on the metal surface, which prevented the enzyme from dissolving in the solution during further experiments. Hence, the lifetime of the sensors in the testing buffer solution was prolonged.

The operation of the lactate oxidase and the destruction of hydrogen peroxide at the anode were based on the chemical reactions below:

(1) $$L‒\mathrm{lactate}+O_{2}\;\underset{\to }{\mathrm{LOx}}\;\mathrm{Pyruvate}+H_{2}O_{2}$$

H_2_O_2_ → HO_2_^•^ + H^+^ + e^‒^ (2)

HO_2_^•^ → O_2_ + H^+^ + e^‒^ (3)

### 2.5. Measurement Procedures

Cyclic voltammetry (CV) experiments were performed to compare the electrical currents flowing through the electrodes of different sizes and the roughness of IrO_x_ and Au thin films. Chronoamperometry were conducted with the electrical current responses recorded by a potentiostat (Pinnacle Tech., Lawrence, KS, USA). The sensor was placed in a beaker with 40 mL 1× PBS with a magnetic rod stirring at the bottom. The temperature was maintained at 37 °C in a water bath to imitate the human body environment. A constant biasing voltage of 0.6 V was applied between the WE and an Ag/AgCl reference electrode (RE) (BASi Inc., Lafayette, IN, USA). Another constant voltage of 0.6 V was applied between the SE and RE with the RE as the common ground. The lactate stock solution was added to the beaker by a succession of 80 µL, which led to a corresponding increased lactate concentration of 2 mM each time inside the beaker. Dopamine and glutamate were applied into the beaker later as the interference molecules to demonstrate sensor selectivity. Scanning electron microscopy (SEM) was conducted to check the enzyme quantity and sensing surface quality before and after the sensor was used.

## 3. Results and Discussion

### 3.1. Cyclic Voltammetry of Sensors and Analyte Detection

#### 3.1.1. CV Characterization on Au Electrodes

**Figure 2:**
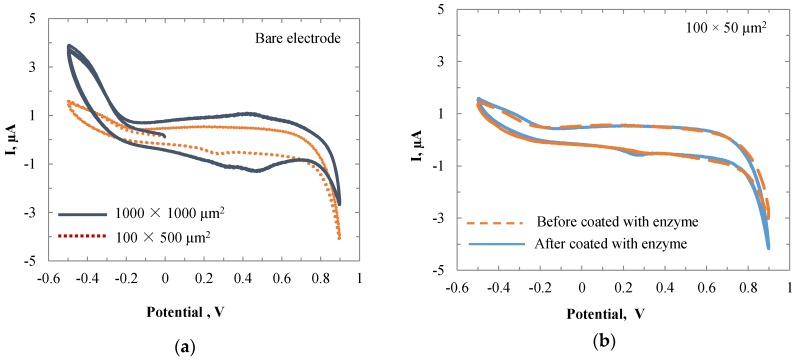
Cyclic voltammograms of (**a**) bare gold electrodes with the sizes of 1000 × 1000 μm and 100 × 50 μm; and (**b**) the sensor with a size of 100 × 50 μm in PBS with 150 mM KCl before and after enzyme coating.

Cyclic voltammetry was performed on Au electrodes with different sizes. The CV experiments were conducted in the 40 mL PBS, 150 mL KCl solution. Figure 2a shows the current-potential (I‒V) curves of Au sensors with the sizes of 1000 × 1000 µm and 100 × 500 µm. It is obvious that with a larger size the I‒V curves were broader, indicating higher current values. Thus, the electrode with the size of 1000 × 1000 µm was expected to have a better performance than the smaller size. The CV tests were also performed on the 100 × 500 µm Au electrode before and after enzyme coating.

#### 3.1.2. CV Characterization on IrO_x_ Electrodes

Increasing the surface roughness to increase the reaction area should lead to an improvement in sensitivity [41]. Cyclic voltammetry was applied to quantitatively analyze the surface roughness of Au and IrO_x_ sensing films. The CV experiments were conducted on the 100 × 50 µm Au and IrO_x_ sensors in the potential window of −0.5 V to +1.0 V in 40 mL 1× PBS with 150 mM KCl. A scanning rate of 300 mV/s was applied.

The roughness factor can be calculated as the ratio of the active reaction area to the geometric area. The functions are shown below:

ρ = A_r_/A_g_ (4)

**Figure 3:**
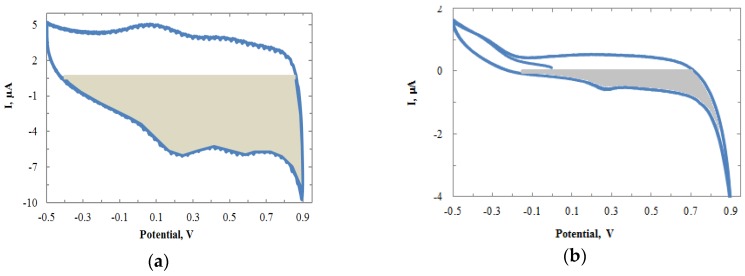
CV plots of (**a**) IrO_x_ film versus an Ag/AgCl electrode in 1× PBS at 300 mV/s; and (**b**) Au film under the same condition. Grey areas were used for calculation of integral regions for Au and IrO_x_.

A_r_ = Q_H_/Q_H_* (5)

where ρ is the roughness factor, A_r_ is the active reaction area, and A_g_ is the geometric area. Q_H_ is the total charge, which can be calculated by taking the integral of the CV curve. Q_H_* is the charge density for the single layer molecules of the substrate surfaces. Based on the literature, IrO_x_ has more charge density than Au [42]. In the experiments, Q_H_* (the absorption of oxygen) of 386 µC/cm^2^ is applied to Au. For IrO_x_, it is in the range of 500‒1900 µC/cm^2^, depending on the film conditions [43,44]. By taking the integral in the CV plots, the Q_H_ was obtained. Figure 3a,b show the integral regions for IrO_x_ and Au (grey area), respectively.

**Figure 4:**
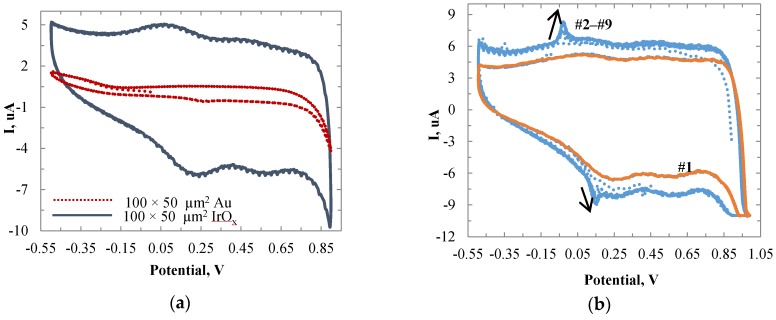
(**a**) CV plots of an Au electrode and IrO_x_ modified electrode with a size of 100 × 50 μm in PBS at 300 mV/s. (**b**) CV plots of the IrO_x_ electrode in PBS at 300 mV/s. Curve #1 was the CV curve before the adding of lactate. Curves #2‒#9 were the respective results after eight successive additions of lactate.

After calculation, the roughness factor ρ for Au was obtained as 0.512. For IrO_x_, Q_H_ by calculation was 5.69 and with Q_H_* in the range of 500‒1900 µC/cm^2^, the ρ was in the range of 1.2‒4.5496. Clearly IrOx had a higher roughness factor and more enzymes could be loaded on the sensing surface. As expected, the IrO_x_ sensing film increased the sensor sensitivity. Figure 4a shows the CV plots for Au and IrO_x_ sensors with the same electrode size of 100 × 50 µm. The result showed the conductivity was greatly improved for the IrO_x_ electrode, with a small reduction peak observed at around 0.19 V.

#### 3.1.3. CV on Titration Tests

Titration tests were conducted with the cyclic voltammetry. Figure 4b shows the CV traces for the IrO_x_ sensor before adding lactate (curve #1) and with eight successive accessions of lactate (curves #2‒#9). Each time, 80 µL lactate stock solution was applied, which led to concentrations of lactate in the beaker increasing from 2 mM to 16 mM. The oxidation peak current at the bias of approximately −0.05 V increased with respect to each addition of lactate, due to the generation of H_2_O_2_ in the enzymatic reaction. The reduction peak at approximately 0.15 V also increased, which was caused by the consequential electrocatalytic reduction of H_2_O_2_ [40]. This phenomenon was only observed on the electrode with LO_x_ enzymes.

### 3.2. Sensitivity Tests

**Figure 5:**
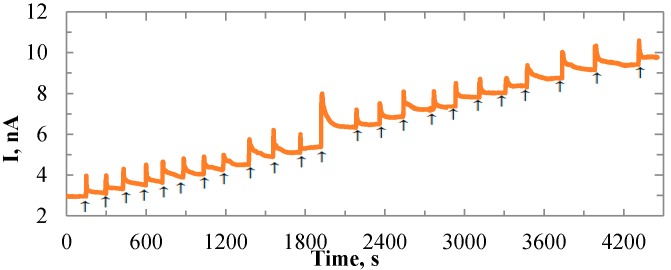
Time-current plot for the enzyme coated Au electrode in 1× PBS with the response to lactate addition. Each addition is 2 mM. The arrows indicate additions of lactate solution.

**Figure 6:**
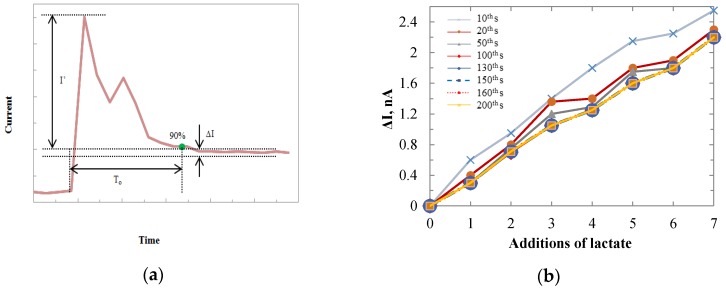
(**a**) Definition for current overshoot (I’), current fluctuation (∆I), and transition time (T_o_). (**b**) Responsive current values at different time points after adding the lactate solution.

**Figure 7:**
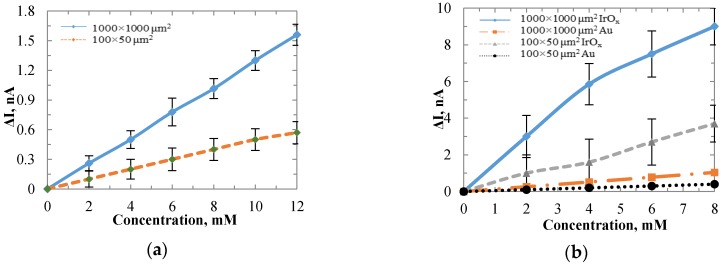
Sensitivity comparison for (**a**) Au electrodes with sensing areas of 1000 × 1000 μm and 100 × 50 μm; and (**b**) Au and IrO_x_ electrodes with areas of 500 × 500 μm and 1000 × 1000 μm.

Chronoamperometry of titration tests was first conducted by the Au sensor with a size of 1000 × 1000 µm. A constant potential of 0.6 V was applied between the WE and Ag/AgCl reference electrode. The performance of the sensors is shown in Figure 5. Successive additions of 2 mM lactate led to corresponding stepwise increases of electrical currents. After 11 additions, a shot of 320 µL lactate was added to the beaker to ensure that the current increases were from the lactate additions. Then another 11 additions of 80 µL lactate were added successively. The increased currents produced at the Au anode in the H_2_O_2_ oxidation process were proportional to the lactate concentrations. During lactate additions, the lactate mixture was dripped closely near the gold sensing surface, and then defused into the buffer solution. The lactate concentrations at the certain dipping time points were much higher until they were diluted in the solution. Hence, overshoots of signals were observed when the lactate was first added. To calibrate the sensor and make a consistent discussion on the stability and sensitivity of electrodes based on different sensing films, we define the performance related terms in Figure 6a. The red curve in Figure 6a imitated the general current change for one addition of lactate. The units for the graph are relative, which will be defined by the researcher in the experiments. The current overshoot phenomenon and the tendency of current transition were presented. The current overshoot (*I’*) was defined as the difference between the peak current value and 90% of the saturated current value. The overshoots ranged from 0.6–1.5 nA in different additions of lactate. The current fluctuation (∆I) was defined as the current variation range after the sensor reached a stable condition. The current fluctuation may be caused by the system noises such as the electrical noises, electromagnetic interferences, vibration of the testing instrument, and liquid dynamics. The current fluctuation was typically less than 0.15 nA. The transition time (T_o_) was defined as the time period from the beginning of the current overshoot until the current reached 90% of the saturated current value. To investigate the transition time of the sensor after lactate has been added, the current values were measured at different time points after each addition of lactate. The results were shown in Figure 6b. The value of the x-axis indicates the number of times that lactate was added. The annotation “Xth s” means the time period from the time lactate was added to the buffer solution until the current value was measured. The current values were taken after the lactate was added to the PBS for 10, 20, 50, 100, 130, 150, 160, and 200 s. Based on the results, after 100 s the sensors showed the same current value for different time points, as the current data points overlapped after the 100th s. Hence, we conclude that the transition time for the Au electrode was 100 s. The same experiment was also conducted for IrO_x_ electrodes, which showed the same result as the Au electrode. With the measured currents at the 100th s after the overshoots, the titration test showed a sensitivity of 129.6 pA/mM. The titration test was also conducted on the 100 × 50 µm Au electrode to investigate the sensitivity for different sensing area sizes. Figure 7a shows the sensitivity comparison between the 100 × 50 µm and 1000 × 1000 µm Au electrodes. For each sensor size up to 20 electrodes were tested to calculate the average sensitivity. By increasing the sensing area, the average sensitivity increased from 47.5 pA/mM to 129.6 pA/mM. Hence, the sensitivity was improved. However the surface area normalized sensitivity dropped from 950 nA/(cm^2^ mM) to 13 nA/(cm^2^ mM). This may be due to the surface tension from the enzyme stock solution on the electrodes. The enzyme mixture was a suspension in which the LO_x_ biomacromolecules were not evenly distributed. The ionic strength of the PBS was interfering with the solubility of the enzyme. After the air-dry process, the proteins most likely located on either the center or the boundary of the solution drop. For the smaller sensing pad, relatively more in terms of percentage of the proteins, were accumulated on the metal. Thus, more current density was produced in a smaller area. This issue may be resolved with robotic suspension to apply the enzyme, which is commonly performed in pharmaceutical practice.

To increase the surface area normalized sensitivity, we modified the electrode surface with IrO_x_. IrO_x_ has a higher roughness factor than gold, which makes it possible to accumulate more enzyme proteins. The sensitivities were compared between Au and IrO_x_ modified electrodes with different sizes. For the electrode with the size of 100 × 50 µm, the sensitivity increased from 47.5 pA/mM to 462.5 pA/mM, the normalized sensitivity increased from 950 nA/(cm^2^ mM) to 9250 nA/(cm^2^ mM). For the electrode with the size of 1000 × 1000 µm, the sensitivity increased from 129.6 pA/mM to 1125 pA/mM , the normalized sensitivity increased from 13 nA/(cm^2^ mM) to 112.5 nA/(cm^2^ mM). The results of the sensitivity comparison were shown in Figure 7b. IrO_x_ increased the surface area normalized sensitivity by 9.17 times for the same sensing size. The surface tension issue of enzyme coating remained as the smaller surface area yields higher normalized sensitivity.

### 3.3. Selectivity Tests

**Figure 8:**
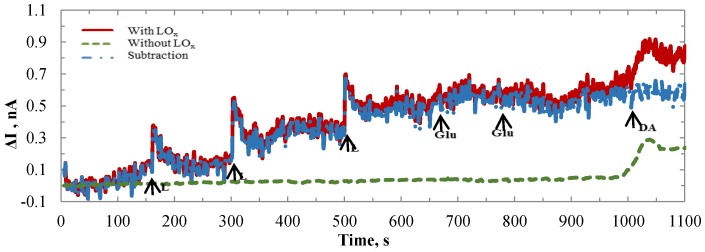
Time-current plots for WE (with LO_x_) and SE (without LO_x_) in 1× PBS with responses to lactate, glutamate, and dopamine.

Glutamate and dopamine were used as the interference bio-molecules. They were applied to the 1000 × 1000 µm Au lactate sensor individually. Figure 8 shows the sensor current responses to lactate, glutamate, and dopamine. Fifty microliters Glu and 10 µL DA were added in turns after three accessions of 80 µL lactate. The baseline currents were different for the WE (with LO_x_) and SE (without LO_x_) because the loaded enzyme changed the impedance of the WE. The baseline currents were recalibrated. Then the SE values were subtracted from those of WE to remove the interference effects which were more noticeable for the DA (on the right side of the green dotted curve). The net values (blue dashed curve) showed that the sensor had no responses to Glu and DA.

**Figure 9:**
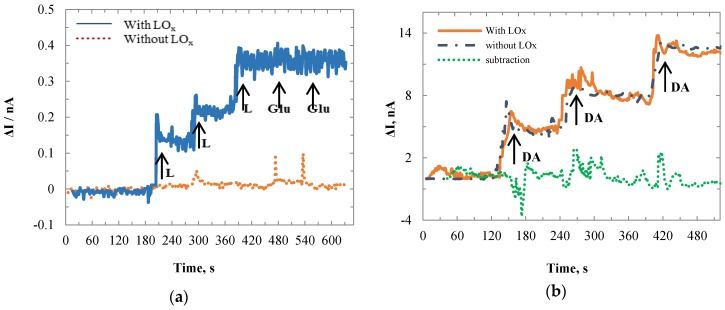
Time-current plots for WE (with LO_x_) and SE (without LO_x_) in 1× PBS with responses to (**a**) lactate and glutamate; and (**b**) dopamine only.

The sensor was removed from the beaker and cleaned by 1× PBS solution. The second and third experiments were conducted separately with respect to Glu and DA. Figure 9a shows the sensor responses to the additions of lactate and glutamate. The LO_x_ enzyme modified WE had corresponding responses to lactate, while the bare Au SE showed no response. Both WE and SE showed no responses to Glu since it was not an electrode reactive component. The two overshoot signals from the SE were induced by the electron turbulence when the Glu was first added to the beaker. The noises in WE were noticeable compared with those in SE. The reason may due to the interference induced by the chemical reaction conducted on the sensing film. Figure 9b shows the sensor response to dopamine. Both WE and SE showed similar responses caused by the oxidation potential of DA on electrodes. The subtracted values (green dotted curve) showed little response to DA. However, some overshoot and disturbance signals were observed, which happened at the time point when the dopamine was added to the buffer solution. Since the working electrode was covered by the lactate enzyme protein while the self-referencing electrode was directly in contact with the dopamine, there is a response time difference between the two electrodes. Additionally, it was difficult to add the DA solution at the exactly equal distances to the two electrodes. Hence, noise was generated when we did the subtraction for the current responses. However after the two electrodes became stabilized, the noise of subtraction reduced. After three additions of dopamine, the current value was still at around 0 nA, same as the initial condition. In the entire time range the subtraction values showed no current increases with respect to the additions of dopamine. Hence, the self-referencing technique can eliminate the interference caused by DA. The selectivity test demonstrated that the sensor probe was responsive only to the additions of lactate.

**Figure 10:**
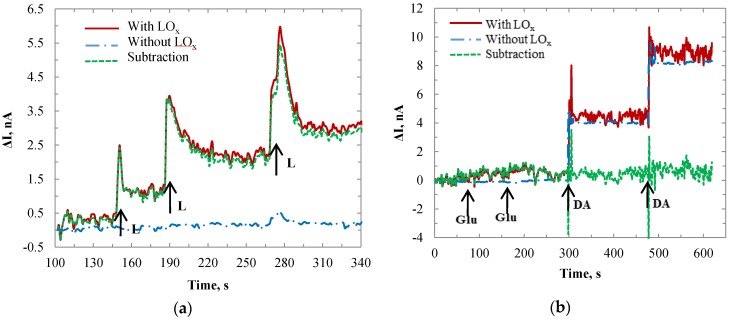
Time-current plot for the IrO_x_ modified WE (with LO_x_) and SE (without LO_x_) in PBS with responses to (**a**) lactate and (**b**) glutamate and dopamine.

The self-referencing technique was also applied to the IrO_x_-modified sensor. Figure 10a shows the current values conducted with a 1000 × 1000 µm IrO_x_ sensor. Similar to the Au sensor, the IrO_x_ modified sensor showed corresponding stepwise increased currents with respect to the additions of lactate. The current step each time was approximately 10 times larger than that of the Au electrode with the same size. Same as the Au electrode, the IrO_x_ modified sensor showed no response to interferences, such as glutamate and dopamine, as shown in Figure 10b.

### 3.4. Longevity Tests

After a few days of use, the Au sensor showed decayed performance. The sensor eventually stopped responding to lactate. This may be due to the loss of weakly-bonded enzymes, the inactivation of the enzyme, or the damage of the sensing film surface. To examine the electrode lifetime in a dry condition, the sensor was first tested in one beaker with four additions of lactate, and then sealed in a container for a week at room temperature. The same experiment was conducted and repeated every week. In this case the sensor showed responses to lactate for four continuous weeks. At the fifth week, the sensor started to show a degraded response with less sensitivity. Scanning electron microscopy (SEM) was conducted to check the enzyme quantity and sensing surface quality before and after the sensor was used.

**Figure 11:**
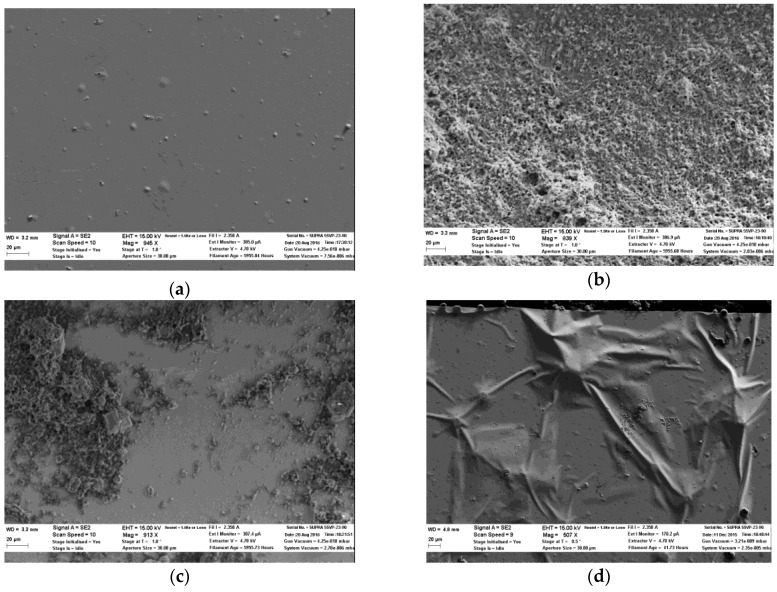
(**a**) SEM photo of the Au film before being loaded with enzymes. (**b**) SEM photo showing the structure of the cured lactate protein on the Au surface. (**c**) SEM photo of the Au sensor after being used for three weeks. The amount of the enzyme protein was decreased. (**d**) SEM photo of the bumpy Au surface after the probe was used for a month.

Figure 11 shows the SEM images of the Au sensing film before and after use. Figure 11a shows the condition of Au film before enzyme was loaded. The entire surface was flat and smooth. The small bumps were caused by the dust particle on the polyimide film before metal deposition. Figure 11b shows the structure of the enzyme protein. It clearly shows the protein was evenly distributed on the flat film. Figure 11c shows the Au sensing surface after being used for a month. The amount of enzyme protein decreased compared with Figure 11b. This was caused by the dissolution of protein in the PBS solution during the experiment. Figure 11d was taken after the Au electrode stopped working. A bumpy Au surface was observed. It was likely that, after several tests, some of the protein particles were washed away by the buffer solution along with the attached Au film, which left micro-scale pores on the metal layer. The buffer solution leaked through the pores and went under the film to create the bumps. Thus, the sensing pad was damaged.

**Figure 12:**
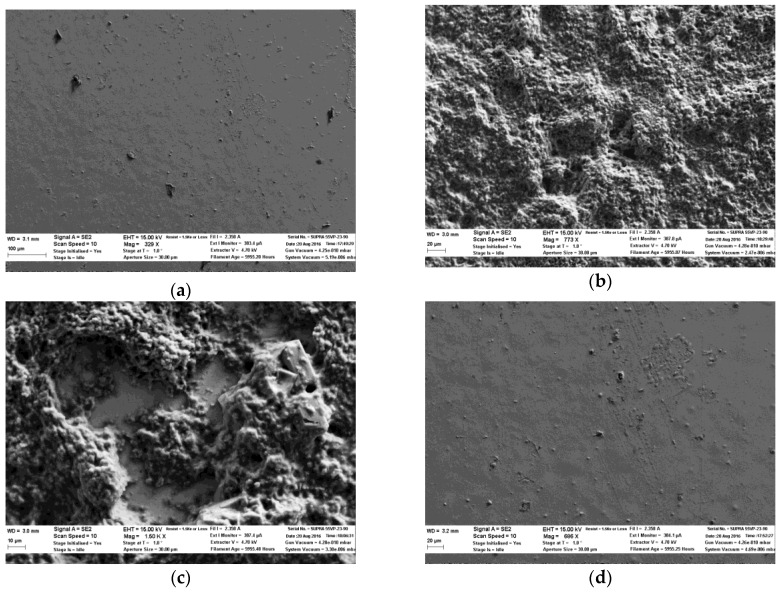
(**a**) SEM photos of IrO_x_ film before loaded with enzymes. (**b**) SEM photo showing the structure of the cured lactate protein on the IrO_x_ surface. (**c**) SEM photo of the IrO_x_ sensor after being used for three weeks. The opening area in the center shows the missing enzyme protein. (**d**) SEM photo of the IrO_x_ surface after the probe was used for a month.

Figure 12 shows the SEM images of the IrOx sensing film before and after use. Figure 12a shows the IrO_x_ sensing surface before being loaded with the enzyme. The bumps indicate the cracks of the IrO_x_ crystal which were generated during the heating process. Figure 12b,c show the structure and distribution of the enzyme protein on the IrO_x_ sensing surface before and after being used for a month. Compared with Figure 11c, more of the enzymes were preserved on the IrO_x_ surface after use. Figure 12d was taken after the enzymes were dissolved. The damage of the Au film was not observed for IrO_x_. Hence, in addition to the increased sensitivity, the rough surface of IrO_x_ allowed better loading of the enzyme and could possibly eliminate gas evolution from the reaction that caused metal corrosion.

### 3.5. Flexiblility Tests

**Figure 13:**
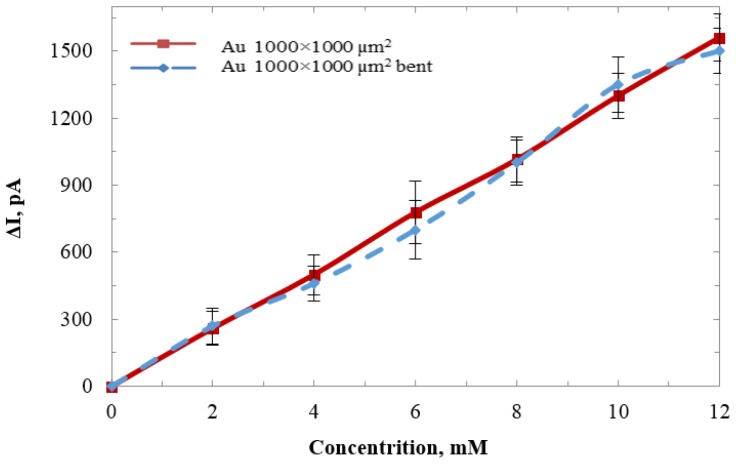
Sensitivity comparison between the sensor in the flat condition and when it was bent.

The lactate sensors are designed suitable for wearables and implants, owing to the flexibility of the substrate. The sensitivity was tested on a 1000 × 1000 µm sensor in flat and bent conditions. The polyimide substrate supporting the electrodes was bent with a curvature radius of 2 mm. A cotton wire was used to tie the probe shaft to keep the sensor in the bent condition. Figure 13 shows the comparison between the sensor in the bent and flat conditions. The result demonstrated that the sensitivity was not affected when the sensor was deformed to a curvature radius of 2 mm. A longer response time was observed during experiments. This was likely due to that the sensing electrode facing inwards in the bent probe, hence the applied lactate was not directly touching the electrode. Time for diffusion was needed before the reaction occurred.

## 4. Conclusions

In this work, a lactate-oxidase-based flexible lactate sensor was developed. Two types of biocompatable electrode films, gold and IrO_x_, as the primary materials for wearable or implantables have been demonstrated. Sensors with different sizes and materials were compared for sensitivity, selectivity, stability, and durability. The rough surface of IrO_x_ provides an improvement in sensitivity. The self-reference technique reduces interference and noise, providing a better selectivity. The simple fabrication method without high thermal budgets provides potentially cost-efficient fabrication of sensors. The flexible polyimide substrates, along with the IrO_x_ electrode being inert, enables the device better biocompatibility for animal and human use. The good performance of the sensing electrodes and the simple fabrication method make an affordable device possible for a variety of practical applications. Disposable devices could be achieved for clinical medicine, food processing, athlete training, and other lactate-detection-related applications.
